# Physician–Patient Language Discordance and Poor Health Outcomes: A Systematic Scoping Review

**DOI:** 10.3389/fpubh.2021.629041

**Published:** 2021-03-19

**Authors:** Naomi Cano-Ibáñez, Yasmin Zolfaghari, Carmen Amezcua-Prieto, Khalid Saeed Khan

**Affiliations:** ^1^Department of Preventive Medicine and Public Health, Faculty of Medicine, University of Granada, Granada, Spain; ^2^Consortium for Biomedical Research in Epidemiology and Public Health (CIBERESP), Granada, Spain; ^3^Instituto de Investigación Biosanitaria ibs. GRANADA, Granada, Spain; ^4^Women's Health Research Unit, Barts and the London School of Medicine and Dentistry, London, United Kingdom

**Keywords:** language concordance, physician, patient, health outcomes, migrants

## Abstract

**Objective:** This systematic review assessed whether physician–patient language concordance, compared with discordance, is associated with better health outcomes.

**Methods:** A systematic literature search was conducted, without language restrictions, using PubMed, EMBASE, Web of Science, and PsycINFO, from inception to July 2020. We included studies that evaluated the effects of physician–patient language concordance on health outcomes. Articles were screened, selected, and data-extracted in duplicate. Review protocol was prospectively registered (PROSPERO, CRD42020157229).

**Results:** There were 541 citations identified through databases and eight citations through reverse search and Google Scholar. A total of 15 articles (84,750 participants) were included reporting outcomes within five domains: diabetes care (four studies), inpatient care (five studies), cancer screening (three studies), healthcare counseling (two studies), and mental health care (one study). Ten studies were of good quality, four were fair, and one was poor, according to the modified Newcastle-Ottawa Scale. Eight studies (53%) showed a significant negative association between language discordance and at least one clinical outcome. Five studies (33%) found no association.

**Conclusion:** Over half the evidence collated showed that physician–patient language concordance was associated with better health clinical outcomes.

## Introduction

In the last decade, around 3.4% of the world population (258 million) has migrated, the majority into the United States (USA) (1). In 2018, 21.8 million of 446.8 million people in the European Union (EU) were non-EU immigrants, equating to ~4.9% of the total population across all 27 countries (2). With an ever-growing number of migrants, a subset of whom include asylum seekers and refugees who face specific healthcare challenges, many also face linguistic obstacles in accessing adequate healthcare services (3).

Language differences between healthcare providers and migrant patients, in addition to restrictive legal status and socioeconomic difficulties, are recognized as a major global healthcare barrier (4). However, the evidence of the negative effect of language discordance on healthcare provision is not completely understood. Language differences have been associated with medication non-compliance, adverse drug events, and underuse of preventative care (5, 6). Interpreters may play a role in addressing language discordance (7), but this intervention is associated with shortcomings including longer waiting times, increased consultation duration, and an increased financial burden on healthcare services (8). Insurance in private healthcare systems may not cover interpreter costs, and, surprisingly, only around half of EU member states provide free interpreting services (9). Thus, alternative approaches in tackling language barriers are sought on a daily basis in health services, to varying effects.

Current literature exploring associations between language barriers and health outcomes, e.g., patient safety, provide an incomplete understanding of the health disparities observed (10). When compared with language discordance in individual studies, physician–patient language concordance has been associated with increased patient follow-up among those affected by chronic illness, avoidance of medication complications, and reduction in the use of emergency care services (11). Previous reviews have reported a positive impact of language concordance on subjective outcomes, such as care satisfaction, access to healthcare, and perceived quality of care. In such cases, it is possible that the positive effects of language concordance have been magnified due to the lack of objectivity. Reviews with laboratory-based health measures have been limited to evaluations of diabetic patients (12). Furthermore, previous reviews appear not to have followed PRISMA reporting guidelines (13). Evaluations using AMSTAR tool (14) have found them to be of poor methodological quality, without an independent prospective registry (11, 12) or an assessment of risk of bias in the studies included (11). To build upon previous findings, to explore the effects across a wider range of healthcare domains, and to focus attention on objective clinical outcomes, we undertook a robust systematic review to comprehensively assess whether physician–patient language concordance, compared to discordance, is associated with improvements in health outcomes.

## Methods

The protocol of the systematic review was prospectively registered in PROSPERO with the registration number CRD42020157229 (https://www.crd.york.ac.uk/PROSPERO). We followed PRISMA reporting guidelines (13). The review question (PICOS), registered in PROSPERO, was as follows: population (P): patients seeking health care; exposure (I): patient–health care provider language discordance; comparison (C): patient–health care provider language concordance; outcome (O): health measurements; and study design (S): systematic review of observational studies.

### Search Strategy and Study Selection

A literature search was carried out in June 2020 across four bibliographic databases: PubMed, PsycINFO, Web of Science, and EMBASE. We did not apply limits to publication date or article language. In order to achieve an optimal search strategy, the following indexing terms, word variants, and free text terms were applied. The MeSH-terms combined in the search strategy were as follows: (“Language discordance” OR “Language concordance” OR “language prof^*^” OR “interprofessional) AND “language” (“health outcomes” OR “clinical outcomes” “diabet^*^” OR “hyperten^*^” OR “cardiovasc^*^” OR “psych^*^” OR “mental health” OR “physical health”) AND (“doctor” OR “physician” OR “clinician”) AND (“patient”). A manual search was also conducted, through reverse search and Google Scholar. We used EndNote bibliographic citation program to pool the articles that were yielded and remove duplicates.

Two reviewers (YZ and NC-I) independently screened the titles and abstracts and reviewed the full texts for study selection. Articles were initially screened by title and abstract and were included if they (1) were observational studies; (2) included an exposure group of patients that were identified as language concordant or discordant with their physicians; (3) identified an association between language discordance and patient health outcomes; and (4) observed health outcomes with objective health measurements, such as cancer screening rates, serum HBA1c levels, post-operative length of stay (POLS), or blood pressure measurements. Articles were excluded if they solely included self-reported measures of health outcomes or other forms of subjective outcome measures, such as patient satisfaction of care, patient experience, or medical comprehension. Articles that only included the use of interpreters and failed to explore physician–patient language concordance specifically were also excluded. Qualitative studies, reports, and gray literature were excluded.

### Data Extraction and Study Quality

Two reviewers (YZ and NC-I) independently extracted data and assessed the selected study quality. The following information was obtained from the articles for analysis: first author, publication year, study locations, study design, sample size, population, type of objective health outcome(s) studied, participants' race and/or ethnicity, languages documented, effect of language concordance/discordance, comparison groups, and main findings. Risk of bias of each study was assessed using the modified Newcastle-Ottawa Scale, which addresses three aspects of quality: (1) selection: representativeness of the exposed participants to language discordant care, selection of the non-exposed participants, and ascertainment of exposure to language discordant care; (2) comparability: confounders and study design; and (3) outcome: assessment of health outcome, adequate retention of cohort (%), and follow-up period (years) (15).

### Study Synthesis

Studies were narratively categorized into five domains depending on the clinical outcome measure: diabetes care, inpatient care, cancer screening, healthcare counseling, and mental health care. We tabulated our findings. Inferences were generated taking study precision and quality into account, since quantitative synthesis (meta-analysis) proved unfeasible due to substantial heterogeneity of exposure, outcome, study quality, and statistical analyses. We settled for a qualitative synthesis in the form of vote counting, which we conducted within broad exposure-outcome subgroups stratified by study quality and precision to minimize bias. Due to the wide range of health outcomes reported across studies, we summarized the direction of the results using a vote-counting approach, quantifying studies on the basis of their positive, negative, or non-significant outcomes. This approach is in line with what is considered suitable (16, 17) to avoid subjectivity.

## Results

**Figure 1:**
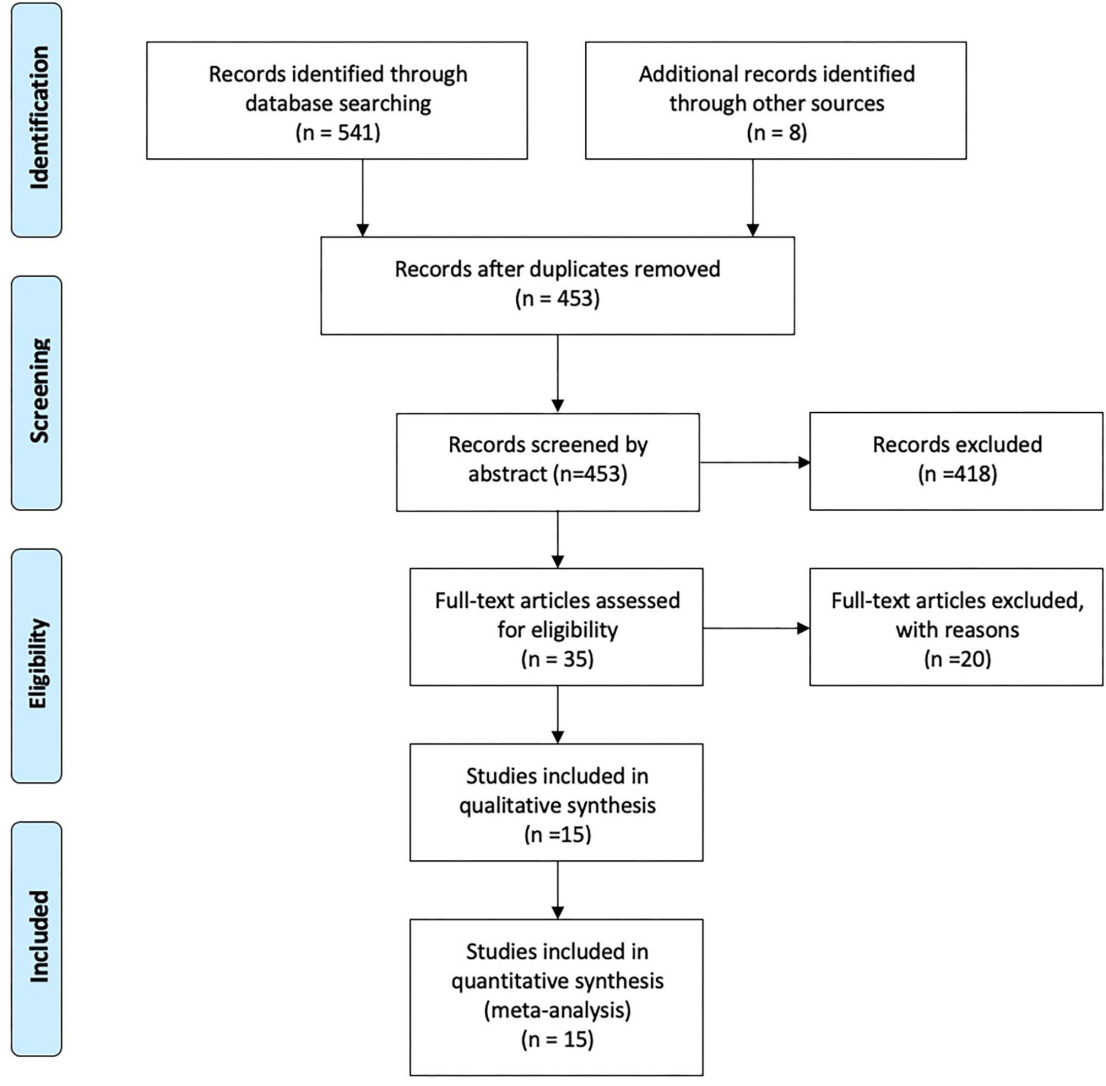
Flow chart. Physician–patient language discordance and poor health outcomes: A systematic review, Spain–UK, 2020.

Our study search yielded 541 citations. An additional eight articles were identified through searching reference lists of relevant studies. After removal of 96 duplicates, 451 articles remained for title and abstract screening. After exclusion of 418 articles that did not meet the selection criteria, 35 studies were carried forward for full-text review and assessed for eligibility. Fifteen out of the 35 studies were eligible, from which data on study characteristics and main findings was extracted (Figure 1).

### Study Characteristics and Quality

Out of the 15 studies, 12 studies (80%) were conducted in the USA, one study (6.7%) in Australia, one study (6.7%) in Canada, and one study was based (6.7%) in Sri Lanka. Ten studies (66.7%) were retrospective cohort studies, four studies (26.7%) were RCTs, and one (6.7%) was a cross-sectional observation study. Sample size ranged from 55 to 44,983 participants. Eight out of 15 studies (53%) focused on only Spanish-speaking patients, almost all of which had a study population of solely Hispanic patients (Table 1). Four out of 15 studies had no mention of using race/ethnicity as control variables in their study at all.

**Table 1:** Study characteristics.

**References**	**Location**	**Study design**	**Sample size**	**Language (s)**	**Patients**	**Race/ethnicity**	**Intervention**	**Health outcomes**
Manson (18)	USA	Retrospective cohort study	96	Spanish; English	Adult Spanish-speaking population with asthma, seen from July 1979 to March 1987	Hispanic	Physician-patient LC vs. LD	Serum levels of theophylline; ER visits for asthma, follow-up appointments
Linsky et al. (19)	USA	Retrospective cohort study	23,297	English; Spanish; Other	US civilians aged 50 or more with no self-reported history of colorectal cancer	White non-Hispanic; Black; Hispanic; Asian	Physician-patient LC vs. LD	Colorectal cancer screening by Fecal Occult Blood Test (FOBT) and endoscopy
Parker et al. (20)	USA	Randomized Control Trial	1,605	Spanish; English	Limited English Proficiency Latino patients with diabetes	Hispanic	Primary care physician's LD vs. LC	(HbA1c) <8%; LDL <100 mg/dL; SBP <140 mm Hg)
Biswas et al. (21)	Australia	Retrospective cohort study	650	Vietnamese; Greek	Patients undergoing primary PCI between 2013 and 2016	Not reported	LEP vs. EP patients with English speaking physicians	Total ischaemic time
Karra et al. (22)	Sri Lanka	Cluster randomized trial	4,497	Sinhala; Tamil	Women who delivered at six hospitals in Sri Lanka between 2015 and 2017	Sinhalese; Non-Sinhalese	Ethnolinguistic concordance vs. Ethnolinguistic discordance	Rates of post-partum counseling
Eamranond et al. (23)	USA	Retrospective cohort study	306	Spanish	Spanish-speaking patients with interpreter services between 2001 and 2006	Not recorded	Patients with LD vs. LC physicians	Counseling on exercise, diet, and smoking.
Eamranond et al. (24)	USA	Retrospective cohort study	306	Spanish	Spanish-speaking patients with interpreter services between 2001 and 2006	Not recorded	Patients with LD vs. LC physicians	Hyperlipidaemia; Cervical cancer; Breast cancer and colorectal screening
Fernandez et al. (25)	USA	Cross-sectional study	6,738	Spanish; English	Limited English Proficiency Latinos with diabetes	Hispanic; White	LEP patients with LD physician vs. LEP patients with LC physicians	Poor glycemic control (HbA1c > 9%)
Inagaki et al. (26)	USA	Retrospective cohort study	324	Albanian; Bosnian; Haitian Creole; Italian; Portuguese; Spanish	Non-English-speaking patients who underwent inguinal bypass for claudication at an urban, academic medical center, between 2007 and 2014	American Indian/Alaska Native; Black or African American; White; Hispanic or Latino	English speaking patient group vs. Non-English-speaking patient group	POLS, 30-day wound infections, 30-day adverse graft events, unplanned readmissions ≤30 days, and return visits ≤30 days
John-Baptiste et al. (27)	Canada	Retrospective cohort study	44,983	English; Portuguese Italian; Chinese, Greek; Polish; Spanish; others	Limited English Proficient inpatients at three hospitals between 1993 and 1999	Not recorded	LEP vs. English Proficient patients	POLS and odds of in-hospital death
Rostanki et al. (28)	USA	Retrospective cohort study	279	English; Spanish; Other	Patients who received tissue plasminogen activator in emergency department between 2011 and 2014	Hispanic/LatinoWhite	LC vs. LD patients	Door to Imaging time (DIT), Imaging to Needle (ITN) Time in thrombolysis pathway
Leng et al. (29)	USA	Randomized Control trial	198	English; Spanish; Mandarin; Cantonese	Speaking immigrants from TB endemic countries, visiting a primary care clinic for the first time between 2003 and 2005	Asian Hispanic/Latino	LC vs. LD patients	Tuberculin testing
Kim et al. (30)	USA	Randomized Control Trial	78	Korean; English	Korean American patients older than 49 years seeking primary care services	Asian- American; White	Korean ethnicity and speaking group vs. Korean ethnicity and ES group vs. White and ES group	Rates of Colorectal cancer screening through self-completed FOBT kits
Mehler et al. (31)	USA	Retrospective cohort study	55	Russian; English	Russian immigrant type 2 patients with diabetes	White	Comparison of outcomes before and after arrival of LC provider	LDL, HbA1c, Blood Pressure (mmHg)
Goncalves et al. (32)	USA	Cohort study	1,328	PortugueseEnglish	Portuguese speaking patients receiving psychiatric care with linguistically competent	White; Black; Hispanic; Other	Linguistical competent Center care vs. usual care	Adequate psychiatric treatment, Emergency Room, Inpatient care

*Physician–patient language discordance and poor health outcomes: a systematic review, Spain–UK, 2020*.

*LC, Language Concordance; LD, Language Discordance; ER, Emergency Room; LDP, Low-density lipoprotein; SBP, Systolic blood pressure control; LEP, Limited English Proficiency; PCI, Percutaneous Coronary Intervention; POLS, Post-operative Length of Stay; TB, Tuberculosis; ES, English Speaking*.

The studies addressed clinical outcomes across an array of areas in healthcare, with several studies using more than one outcome measure. Three studies focused on glycemic control in diabetic patients as an outcome measure, all of which used serum HbA1c level measurements (20, 25, 31). Three studies measured rates of colorectal cancer screening (19, 24, 30), two studies measured the number of emergency room visits (18, 26), two studies measured POLS (26, 27), two studies used in-hospital time to treatment (27, 33), and two studies used receipt of lifestyle counseling as their outcome measure (22, 23). Finally, one study focused on the effect of language concordance on the receipt of adequate psychiatric care (32).

All 15 studies were assessed for quality using the modified Newcastle-Ottawa Scale (Table 2). Ten studies were of good quality, four studies were of fair quality (28, 29, 31, 32), and one study was of poor quality (30), with positive, negative, or no differences in health outcomes for the language discordance compared to the language concordance group (Supplementary Figure).

**Table 2:** Quality assessment (modified Newcastle-Ottawa Assessment Scale).

	**Selection**				**Comparability**	**Outcomes**			**Quality score**
**References**	**Representativeness of exposed cohort**	**Selection of non-exposed!!break cohort**	**Ascertainment of exposure**	**Outcome not at start of study**	**Comparability of cohorts**	**Assessment of outcome**	**Length of follow up**	**Adequacy of follow up**	
Manson (18)	Not truly representative of asthmatic patients and the sample size was small	Yes*	Patient language status from chart notes or estated by their physicians*	Yes*	Adjusted by Age, gender, pay-status, disease severity**	Electronic medical record*	Yes*	>40%*	Good
Linsky et al. (19)	Representative of non-English speaking patients that visit primary care services*	Yes*	Language from Survey and Self-administered Questionnaire*	Yes*	Race/ethnicity, age, education, marital status, family income, employment**	Self-reported rates of FOBT and endoscopy	Yes*	No loss of data*	Good
Parker et al. (20)	Representative of LEP Latinos with type 2 diabetes*	Yes*	Spanish speaking physicians if they self-reported “high” fluency in Spanish or without the aid of interpreters*	Yes*	Age, sex, economic race/ethnicity**	Electronic medical record*	*Yes	No loss of data*	Good
Biswas et al. (21)	Representative of patients undergoing Percutaneous Coronary Intervention in Melbourne, Australia*	Yes*	Language other than English as their primary language were defined as having LEP	Yes*	Adjusted by age, sex, BMI, Comorbidities hospital long stay, mortality**	Total ischaemic time of participants was accessed through an electronic database*	Yes*	No loss of data*	Good
Karra et al. (22)	Representative of post-partum women in Sri Lanka as data was collected from 6 major hospitals*	Yes*	Questionnaires distributed to women by investigators	Yes*	Adjusted by number of live births, woman's maternal education and work**	Self-reports of receipt of Post-partum counseling	Yes*	62% of women follow up*	Good
Eamranond et al. (23)	Representative of Latino adult patients as the sample was selected from a two large primary care facilities*	Yes*	Data was accessed through an electronic medical record*	Yes*	Adjusted by age, sex, number of visits**	Electronically documented lifestyle counseling *	Yes*	No loss of data*	Good
Eamranond et al. (24)	Representative of Latino adult patients as the sample was selected from a two large primary care facilities*	Yes*	Data was accessed through an electronic medical record*	Yes*	Adjusted by age, sex, number of primary care visits**	Records of screening for hyperlipidaemia, diabetes, cervical, breast and colorectal cancer*	Yes*	No loss of data*	Good
Fernandez et al. (25)	Representative of Latinos with type 2 diabetes as data was collected from a diabetes registry*	Yes*	Self-completed survey in 5 languages *via* mail, web or phone	Yes*	Adjusted by age, sex, education, incomes, years with DM, Hba1c, Comorbidity **	Laboratory results obtained during routine clinical care*	Yes*	No loss of data*	Good
Inagaki et al. (26)	Representative of patients who have undergone inguinal bypass, collected from a large medical center over a 7-year period*	Yes*	LC was collected using electronic medical records*	Yes*	Age, gender, race, ethnicity, BMI, medical comorbidities smoking status and insurance status **	Participants identified via the electronic medical records using relevant terminology codes*	Yes*	No loss of data*	Good
John-Baptiste et al. (27)	Representative of Canadian inpatients, sample was collected across 3 large Canadian hospitals over a 6-year period*	Yes*	LC collected from an electronic patient information system*	Yes*	Age, sex, marital status, comorbid diagnosis codes, language and Charlson score**	Canadian Institute for Health Information discharge abstract database*	Yes*	No loss of data*	Good
Rostanki et al. (28)	No representative of patients receiving thrombolysis treatment post-stroke	Yes*	Primary language determined by self-report, via questionnaire	Yes*	Yes**	The electronic medical record was reviewed for all patients with treatment*	Yes*	No loss of data*	Fair
Leng et al. (29)	Participants are not representative of Latino and Asian immigrants	Yes*	All participants completed a demographic questionnaire	Yes*	Age, education, year of migration, primary language, or self-reported health status assessed**	Clinical data were abstracted from medical records*	Yes*	Only 17 of the 191 (8.9%) patients were referred for tuberculin testing.	Fair
Kim et al.(30)	Representative of Korean Americans, recruited through Korean community-based organizations *	Yes*	Bilingual pre- and post-survey	Yes*	–	No-cost FOBT kit at the end of the session, and had 4 weeks to mail back the FOBT kit	–	–	Poor
Mehler et al. (31)	Study Participants were not representative of Russian patients with diabetes	Yes*	LC was not accurately in English	Yes*	Non adjusted by control variables.	Hba1c and Lipid panels accessed through the administrative database*	Yes*	No loss of data*	Fair
Goncalves et al. (32)	No representative of Portuguese speaking psychiatric patients in the USA	Yes*	LC: any patient with Portuguese as primary language and mental health visit*	–	Age, marital status, race/ethnicity sex; and diagnosis of mental disorder**	Electronic database*	Yes*	No loss of data*	Fair

*Physician–patient language discordance and poor health outcomes: A systematic review, Spain–UK, 2020*.

*LC, Language Concordance; FOBT, Fecal Occult Blood Test; LEP, Limited English Proficient; DM, Diabetes Mellitus. A “good” quality score required 3 or 4 stars in selection, 1 or 2 stars in comparability, and 2 or 3 stars in outcomes. A “fair” quality score required 2 stars in selection, 1 or 2 stars in comparability, and 2 or 3 stars in outcomes. A “poor” quality score reflected 0 or 1 star(s) in selection, or 0 stars in comparability, or 0 or 1 star(s) in outcomes*.

### Assessment of Language Concordance

The parameters of language concordance were heterogeneous across the studies, with several studies using ethnolinguistic concordance as their measure of exposure as opposed to language concordance alone. Several studies did not specify their parameters of language concordance but rather inferred it based on patients' English language proficiency and the assumption that healthcare consultations were in English. Other studies, however, had wholly inaccurate definitions of language concordance, such as Mehler et al. (31), who used patients' ethnicity and immigrant status as a proxy for limited English proficiency (LEP). Similarly, Biswas et al. (21) defined LEP patients as any participant who did not self-report English as their primary language, negating the existence of multilingual participants. The majority of studies used self-reported surveys and questionnaires to collect data on preferred language and language fluency among patients and physicians, while other studies used electronic medical databases to access patients' primary language(s).

The main findings from each study are summarized in Table 3. Compared to concordance, eight studies (53%) showed a significant negative association between language discordance and at least one measure of clinical outcome among study participants (20, 22, 23, 25, 27, 31–33). Five studies (33%) found no significant association between language discordance and the main clinical outcome (18, 19, 26, 28, 29). Two studies (13%) found a positive association between language discordance and clinical outcomes (24, 30). All three studies that examined diabetes showed statistically significant improvement in glycemic control (HbA_1c_ <8%) in the language-concordant groups compared to their language discordant counterparts (20, 25, 31). Two studies also included LDL control as an additional outcome measure for diabetes care (20, 31), both of which also noted significant improvement following language concordance as an intervention. Four studies examined the effects of language discordance and different measures of in-hospital treatment, inclusive of POLS, 30-day mortality, and time to treatment. Three studies showed no association between language concordance and rates of mortality (18, 26, 33).

**Table 3:** Results of studies.

**Primary author, publication date, location**	**Outcomes**	**Measures**	**Language discordance (LD) vs. language concordance (LC)**	**Relative risk (RR)/odds ratio (OR)/mean/prevalence**	**95% Confidence intervals (CI)/interquartile range (IQR)**	***P-*value**
Manson, et al. (18), United States	Medication adherence to asthma inhalers	Non-therapeutic serum theophylline levels (10–20 mg/dl)	LD = 59% LC = 50%	OR = 1.72	CI: 0.69–4.30	0.24
	Emergency room (ER) use	Number of ER attendance	LD = 48% LC = 55%	OR = 2.07		0.12
	Hospital admissions	Number of hospital admissions	LD = 26% LC = 32%			
	Miss a medical appointment	Non-medical attendance (<8 office visits)		OR = 1.66	CI: 0.86–3.20	0.13
Linsky et al. (19),United States	Use of colorectal cancer screening	Self-reported rates of FOBT and endoscopy	EC = 50.8% LD = 37.9% LC = 28.9%	LC OR = 0.57LD OR = 0.84	CI: 0.46–0.71 CI:0.58–1.2	
Parker et al. (20), United States	Glycated hemoglobin (HbA1c)	Control (HbA1c <8%)	LD = 63% LC = 68%			<0.05
	Glycated hemoglobin (HbA1c)	Poor control (HbA1c >9%)	LD = 21% LC = 18%			
	Low-density lipoprotein (LDL)	LDL control (LDL <100 mg/dL)	LD = 65% LC = 76%			0.03
	Systolic blood pressure (SBP)	Pressure control (SBP <140 mmHg)	LD = 78% LC = 84%			
Biswas et al. (21), Australia	Door-to-balloon time	Time in minutes	LD = 71 min LC = 68 min		IQR: 48–112 IQR: 44–103	0.21
	Total ischaemic time	Time in minutes from symptom onset to first balloon inflation in a coronary artery	LD = 281 min LC = 203 min		IQR: 160–720 IQR: 150–350	0.01
	Median symptom-to-door time			OR = 1.63	CI: 1.05–2.54	0.03
	Median length of hospital stays	Time in days	3 days, equal between LD and LC			0.70
	Major adverse cardiac events (MACE)		LD = 7.1% LC = 5.8%			0.61
	Mortality	Time in days (30 days)	LD = 9.1% LC = 7.8%			0.69
	Unplanned readmissions	Time in days (30 days)	LD = 9.1% LC = 9.7%			0.85
Karra et al. (22), India	Receipt of post-partum contraception counseling (PPIUD)	Received at least one advice of family planning counseling		OR= 0.548	CI: 0.406, 0.738	
Eamranond et al. (23), United States	Receipt of exercise counseling	Having documented counseling for exercise directly related to overall cardiovascular health	LD = 43% LC = 62%	OR = 2.2	CI: 1.4, 3.7	0.002
	Receipt of diet and counseling	Having documented counseling for diet directly related to overall cardiovascular health	LD = 44% LC = 70%	OR = 2.1	CI: 1.3, 3.5	0.005
	Receipt of smoking counseling	Having documented counseling for smoking directly related to overall cardiovascular health	LD = 57% LC = 63%	OR = 1.3	CI: 0.8, 2.1	0.36
Eamranond et al. (24), United States	Hyperlipidaemia	Lipid profile within last five years	LD = 92% LC = 95%	RR = 1.04	CI: 0.76, 1.31	
	Diabetes	Fasting glucose (or a normal random glucose) within last 3 years	LD = 92% LC = 93%	RR = 1.01	CI: 0.76, 1.27	
	Cervical Cancer	Pap smear within last 3 years	LD = 74% LC = 76%	RR = 1.02	CI = 0.72, 1.32	
	Breast Cancer	Mammogram within last 2 years	LD = 87% LC = 89%	RR = 1.01	CI = 0.72, 1.30	
	Colorectal Cancer	FOBT, sigmoidoscopy, barium enema, a/o colonoscopy	LD = 72% LC = 47%	RR = 0.78	CI = 0.61, 0.99	
Fernández et al. (25), United States	Glycaemic control	Poor Glycaemic control (HbA1c >9%)	LD = 27.8% LC = 16.1%	OR = 1.98	CI = 1.03, 3.80	0.04
Inagaki, 2017, United States	Post-operative hospital length of stay after non-emergent inguinal bypass	Mean Hospital days	LD = 11.2 LC = 9.4	AdjustedMean ratio = 1.02	CI = 0.85, 1.23	0.133
		Wound infection	LD = 31.4% LC = 25.7%	OR = 1.87	CI = 0.90, 3.88	0.095
		Adverse graft events	LD = 31.4% LC = 29.0%	OR= 1.23	CI= 0.62, 2.45	0.556
		Readmission	LD = 25.5% LC = 20.4%	OR = 1.51	CI = 0.77, 2.95	0.478
		ED return visits	LD = 23.5% LC = 27.1%	OR = 1.28	CI = 0.58, 2.832	0.546
John-Baptiste, 2004, Canada	Length of stay (LOS) stroke	Time in days	LD = 26.1 LC = 14.9	RR = 1.29	CI: 1.18–1.42	
	Length of stay diabetes	Time in days	LD = 11.6 LC = 7.3	RR = 1.28	CI: 1.13–1.45	
	In—hospital mortality craniotomy procedures	Mortality rate	LD = 10.8% LC = 4.4%	OR = 1.98	CI: 1.34–2.94	
Leng, 2011, USA	Tuberculosis diagnostic	Received tuberculin testing	LD = 7% LC = 10%			0.40
Rostanki et al. (28), USA	Time to thrombolysis in Acute Ischemic Stroke	Door to imaging (DIT) time	LD = 25 LC = 24	Median		0.5
		Imaging to Needle (ITN) time	LD = 30 LC = 33	Median		0.3
		Door- to-needle (DTN) time	LD = 55 LC = 58	Median		0.1
Kim et al. (30), USA	Colorectal cancer screening (CCS)	Fecal occult blood test (FOBT)	LD = 66.2% LC = 49.3%			0.016
Mehler, 2004, USA	LDL	Mg/dl	LD = 126 (34.6) LC = 102 (31.9)	Mean (SD)		0.0002
	HbA1c	%	LD = 8.4 (1.5) LC = 8.0 (1.6)	Mean (SD)		0.007
	Diastolic BP	mm Hg	LD = 82.7 (11.0) LC = 76.3 (11.0)	Mean (SD)		0.0002
	Systolic BP	mm Hg	LD = 143.2 (22.6) LC = 140.6 (20.2)	Mean (SD)		0.3
Goncalves 2013, USA	Adequate care 8 mental health visits	%	LD = 30.4 LC = 58.5	Mean difference (28%)		<0.05

*Physician–patient language discordance and poor health outcomes: A systematic review, Spain–UK, 2020*.

*LD, Language Discordance; LC, Language Concordance; EC, English Concordance; FOBT, Fecal Occult Blood Test*.

Three studies examined the effects of language concordance and cancer screening (19, 24, 30). Surprisingly, two out of the three studies found that language concordance actually lead to lower rates of colorectal screening (24). No significant association was found between language concordance and rates of cervical cancer and breast cancer screening (24, 30). Two aspects of healthcare counseling were assessed across two studies: lifestyle counseling (23) and post-partum contraception counseling (22). Language concordance was found to have significant association on the receipt of lifestyle counseling for diet and exercise. However, there was no association between language concordance and receipt of post-partum IUD counseling (22); however, the study noted significant effect from ethnic concordance.

One study focused on the effects of language concordance on the rates of adequate psychiatric care (eight or more psychotherapy visits), in addition to psychiatric-related ER use among participants. Language concordance results in higher rates of adequate psychiatric care in Portuguese-speaking patients (32). However, no association was found between language concordance and ER use (18).

## Discussion

Over half the evidence collated showed that physician–patient language concordance was associated with better health outcomes. Although this review points to the positive effects of language concordance on objective health outcomes in specialisms such as asthma management, mental health, and glycemic control (HBA1c), based on our findings, we deduce that caution is needed in interpretation of findings. The effect of language concordance in improving health outcomes merits consideration.

Using comprehensive searches to capture all published literature and employing robust quality assessments to limit bias, our review provided positive, negative, or no associations regarding physician–patient language discordance and clinical outcomes. This is an important topic, as the proportion of migrant population in the world and in European countries is expected to rise in the next few years. This systematic review included 15 individual studies with a large sample size overall and with mostly good quality evidence. Thus, our review meets the criteria for a high-quality evidence synthesis.

There were several limitations to this review also. Firstly, because most studies are from the USA, we cannot generalize findings to other contexts. USA was overrepresented, being the study location of 12/15 studies. None of the studies were conducted in Europe, which faces the most rapidly increasing influx of migrants in need of language concordant care. Furthermore, only one study (22) was conducted in a non-Western setting, and thus, we were unable to assess any cross-cultural differences in the effects of language concordance. There was little variation in the languages studied, with most studies focusing on Spanish-speaking and English-speaking patients. These features may affect the generalizability of our findings, despite the fact that English and Spanish are the most widely spoken languages worldwide (34). Half of the studies included in this review were made up of only Hispanic patients. According to the 2018 United States Census Bureau, Hispanic people in the United States are 18.3% of total population. Many of them are undocumented, lack health care coverage, and face many stereotypes, as well as disadvantages that would also affect their health, access to health, and behavior of medical care toward them.

Although most studies included were of good quality, there was heterogeneity in the definitions of language concordance across the studies, making it difficult to ascertain its effects. Also, self-reported data in the studies may affect the validity of the methodology, as it may lead to physicians inaccurately reporting their own fluency. Additionally, using electronic patient records to collect data on language and patients' race/ethnicity means relying on the accuracy of previous documentation from healthcare administrative staff, which may result in information bias. We did not examine the effects of sex and gender in our results, which may affect sex-specific outcomes such as rates of cervical cancer screening and contraceptive counseling (35). Finally, 2/15 studies included were conducted by the same author (23, 24) and used the same sample of patients, leading to the potential for bias with regard to the outcomes of the subsequent study.

### Implications

Two of our studies found that language concordance negatively impacted the rates of colorectal cancer screening, both of which focused on Spanish-speaking populations (19, 24). This finding may have resulted from the presence of higher rates of informed consent among language-concordant groups who may not have fully understood the process of colorectal cancer screening, which can involve invasive procedures such as colonoscopies or endoscopies, without the intervention of language concordant care. Contrastingly, the study by Kim et al. (30), which focused on a Korean-American population, demonstrated an increased uptake of colorectal cancer screening in the language-concordant group, perhaps pointing to the covert influences of ethnicity in rates of colorectal cancer screening. Previous studies have highlighted a pattern of reduced rates of colorectal cancer screening among Latinos, comparative to the general population. Indeed, several studies suggest that educational interventions targeting healthcare literacy within this minority group, as opposed to language concordance, may address this disparity and increase intent to access screening (36).

Interestingly, four studies did not include race or ethnicity as variables in their methodology at all, limiting their results. Our review revealed a potential superiority of ethnic concordance over language concordance in its effects on health outcomes in areas such as mental health or post-partum contraceptive counseling (22). Furthermore, previous studies have suggested that doctor–patient race concordance substantially impacts utilization of healthcare services and communication, reinforcing the need for studies to acknowledge race and ethnicity as potential confounding factors (37). A growing body of evidence appears to suggest that clinician–patient language concordance results in greater developments in patient-centered care, as it minimizes communication errors while concomitantly increasing rapport and cementing trust within the clinician–patient relationship. In turn, clinician–patient language concordance may serve as a cost-saving intervention by reducing the need for an interpreter and thus appointment waiting times, which are often longer for language-appropriate consultations. More effort and time should be devoted to improving the understanding of diagnoses, treatments, and follow-up among patients who experience language-discordant care. In addition, self-report surveys should be developed that assess the understanding of patients in their care plans, so that health professionals become aware of the degree of understanding of their patients and thus adapt their communication toward them. However, there is insufficient literature to assess the acceptability and success of physician–patient language concordance consultations without the presence of interpreters.

### Future Research

There is an obvious need for more prospective studies to be conducted to assess the effects of language concordance on health outcomes, with a stronger focus on ensuring accurate measures of language concordance and physician fluency within their methodologies, particularly in studies focusing on non-English language concordance. Furthermore, the majority of current studies assessing LEP patients focus on Spanish language, which is not reflective of all global migrant populations in need of language-concordant care. Although Spanish-speaking migrants form the predominant migrant population in USA, the current migrant crisis has led to many fleeing conflict zones and has resulted in a huge influx of Syrian, Afghani, and Iraqi migrants into the EU, all of whom are understudied and are likely to substantially benefit from language concordant care (2).

### Conclusions

Overall, this systematic review highlights that among other issues concerning the health of minority groups, attention to language concordance issue is vital in ensuring optimal patient-centered care. Healthcare professionals must appreciate the importance of improving healthcare literacy, as well as recognizing the influences of race, ethnicity, and gender upon health outcomes. With regard to policymaking, we conclude that there is a need for a wholistic strategy that targets migrant healthcare needs, with a higher focus on linguistic and cultural competence across global healthcare systems and a move toward diversity-competent care.

## Author Contributions

All authors listed have made a substantial, direct and intellectual contribution to the work, and approved it for publication.

## Conflict of Interest

The authors declare that the research was conducted in the absence of any commercial or financial relationships that could be construed as a potential conflict of interest.
