# Supplementary figures and images for: Physician–Patient Language Discordance and Poor Health Outcomes: A Systematic Scoping Review
Source: Front Public Health. 2021 Mar 19;9:629041. doi: 10.3389/fpubh.2021.629041 (PMC8017287; doi:10.3389/fpubh.2021.629041)

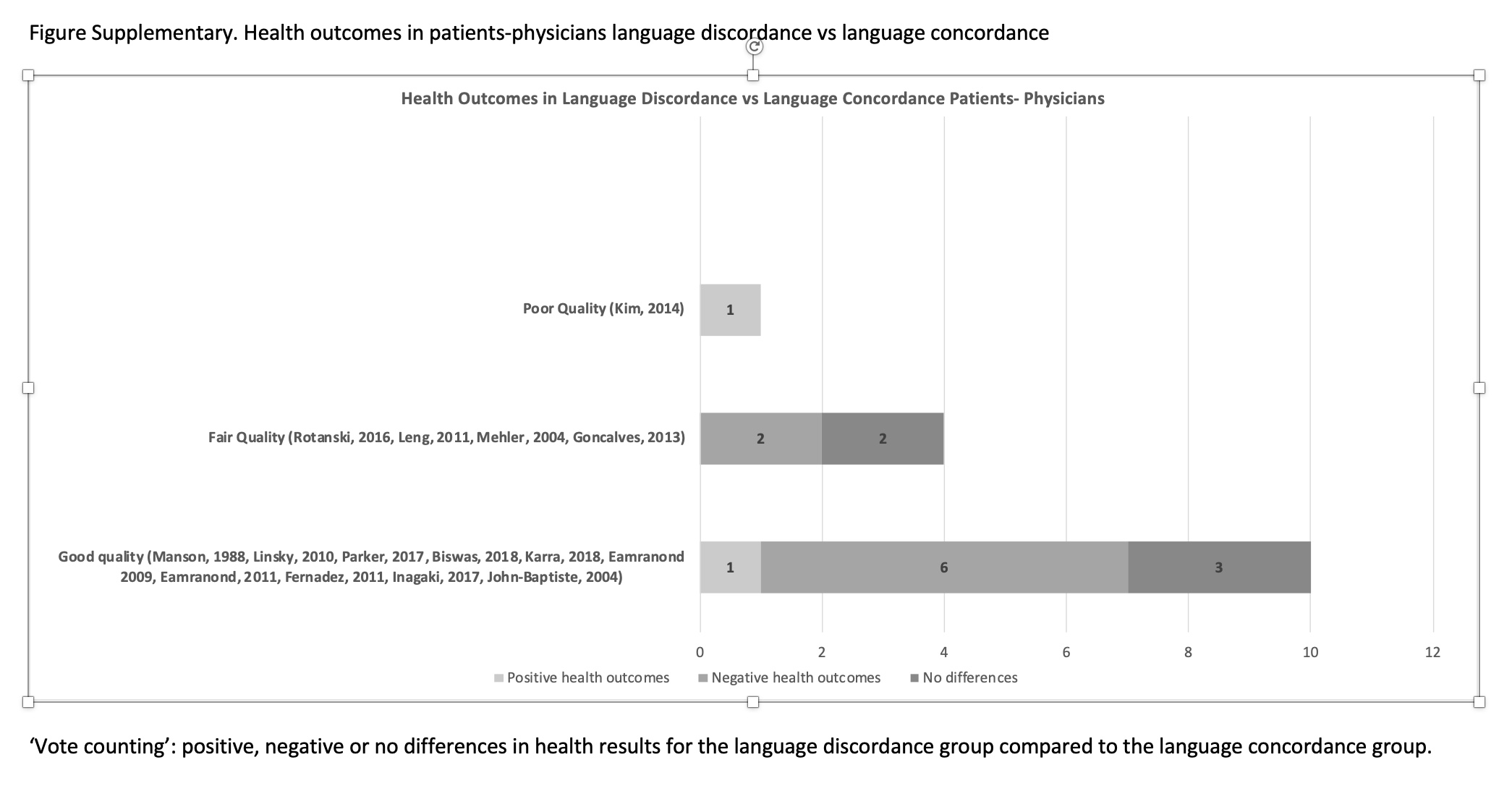

Supplement: Supplementary file 1 [file Image_1.JPEG]
